# Design and development of a magnetic field-enabled platform for delivering polymer-coated iron oxide nanoparticles to breast cancer cells

**DOI:** 10.1016/j.mex.2023.102318

**Published:** 2023-08-05

**Authors:** Bryan Paul Bulatao, Nonthaneth Nalinratana, Pongsakorn Jantaratana, Opa Vajragupta, Pornchai Rojsitthisak, Pranee Rojsitthisak

**Affiliations:** a Center of Excellence in Natural Products for Ageing and Chronic Diseases, Chulalongkorn University, Bangkok 10330, Thailand; b Department of Industrial Pharmacy, College of Pharmacy, University of the Philippines Manila, Manila 1000, Philippines; c Department of Pharmacology and Physiology, Faculty of Pharmaceutical Sciences, Chulalongkorn University, Bangkok 10330, Thailand; d Department of Physics, Faculty of Science, Kasetsart University, Bangkok 10900, Thailand; e Molecular Probes for Imaging Research Network, Faculty of Pharmaceutical Sciences, Chulalongkorn University, Bangkok 10330, Thailand; f Department of Food and Pharmaceutical Chemistry, Faculty of Pharmaceutical Sciences, Chulalongkorn University, Bangkok 10330, Thailand; g Metallurgy and Materials Science Research Institute, Chulalongkorn University, Bangkok 10330, Thailand

**Keywords:** • Construction of the platform for the permanent magnets and 96-well plates, • Fabrication of the CS/Alg-IONPs, • Cytotoxicity evaluation of the CS/Alg-IONPs toward MCF-7 breast cancer cells, Stimuli-responsive, Magnetic targeting delivery, Iron oxide nanoparticles, Alginate, Chitosan

## Abstract

The current literature mostly contains relatively vague descriptions of techniques for implementing *in vitro* magnetic targeting delivery of iron oxide nanoparticles (IONPs), leading to irreproducible processes and incomparable findings. This discrepancy often arises from the varying exposure of IONPs to the non-uniform magnetic field and differences in the concentration of the polymer-coated IONPs. Hence, we meticulously designed and built a system comprising a platform constructed from polyoxymethylene sheets, which securely holds the permanent magnets, and the cell culture plate. We also tailored the preparation process of the IONPs and the *in vitro* toxicity studies. The inherent characteristics of IONPs are further enhanced by their coating with natural polymers, alginate (Alg) and chitosan (CS).

• The design and construction of the platform were carried out using a laser engraving/cutting machine along with graphic design software. The precise locations of the permanent magnets relative to the cell culture plate were determined via a Gaussmeter.
• The quantities of the components in the formulation and the method for fabricating the CS/Alg-coated IONPs (CS/Alg-IONPs) were optimized to ensure that the desired physicochemical properties were obtained.
• The cultivation and cytotoxicity evaluation of the fabricated CS/Alg-IONPs against MCF-7 breast cancer cells were described.

## Graphical abstract

**Figure:**
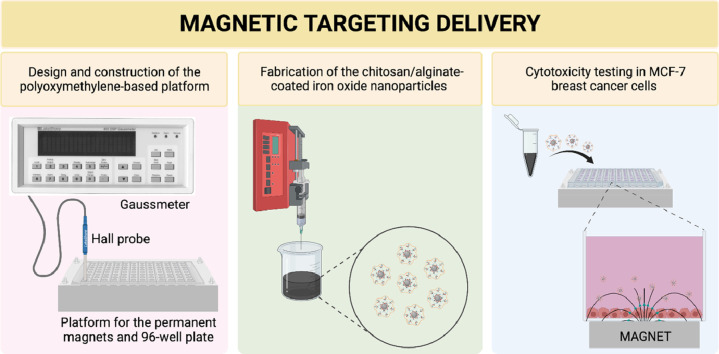

Specifications table

**Table:** 

Subject area:	Materials Science
More specific subject area:	*Magnetic targeting delivery*
Name of your method:	• *Construction of the platform for the permanent magnets and 96-well plates* • *Fabrication of the CS/Alg-IONPs* • *Cytotoxicity evaluation of the CS/Alg-IONPs toward MCF-7 breast cancer cells*
Name and reference of the original method:	*Bulatao, B.P., Nalinratana, N., Jantaratana, P., Vajragupta, O., Rojsitthisak, P., & Rojsitthisak, P. (2023). Lutein-loaded chitosan/alginate-coated Fe_3_O_4_ nanoparticles as effective targeted carriers for breast cancer treatment. International Journal of Biological Macromolecules, 242 (Part 1), 1–15.* doi:10.1016/j.ijbiomac.2023.124673 *Kamiloglu, S., Sari, G., Ozdal, T., & Capanoglu, E. (2020). Guidelines for cell viability assays. Food Frontiers, 1(3), 332–349.* doi:10.1002/fft2.44
Resource availability:	*N/A*

## Method details

Construction of the platform for the permanent magnets and 96-well plates

### Materials

**Note**: The materials are listed in the sequence in which they appear in the procedure.

• Computer with CorelDRAW and CorelLASER programs installed
• Polyoxymethylene (POM) sheets, white, 100 × 150 × 5 mm^3^ (KRE Technology, Navanakorn Industrial Estate, Khlong Luang, Pathum Thani, Thailand)
• Laser engraving machine (LRT-2030, Idea Maker Technology Co., Ltd, Samut Prakan, Thailand)
• Vernier caliper
• Gaussmeter (Lakeshore 455 DSP-Gaussmeter, Lake Shore Cryotronics, Inc., Westerville, OH, USA) equipped with Hall probe (Model HMMA-2502-VR)
• 96-Well, Clear, Flat-Bottom Polystyrene TC-Treated Microplate (Costar^Ⓡ^, Corning Incorporated-Life Sciences, Jiangsu Province, China) Product No. 3599
• Permanent magnets, N35 NdFeB round magnets, 5 × 3 mm^2^ (diameter × length) (Ningbo Townsun Magnet Co., Gaoqiao Industrial Development Area, Yinzhou District, Ningbo, China)
• CSK Philip machine screws, 2 × 22 mm^2^ (diameter × length,) and hex lock nut

### Procedure

#### 1. Platform design using graphic design software

**Note:** POM sheets are selected as construction materials for our platform designed to anchor the permanent magnets and 96-well plate. POM sheets are advantageous for laser cutting since they neither emit acids nor toxic fumes. Their excellent dimensional stability during high-precision cutting and glossy surface finish make them ideal for our purposes. Furthermore, their high resistance to chemicals and melting point of 183 °C render them suitable for sterilization through autoclaving. The platform, comprising three interconnected layers via machine screws, ensures optimal stability and manageability, with each layer being 5 mm thick.

**Fig. 1:**
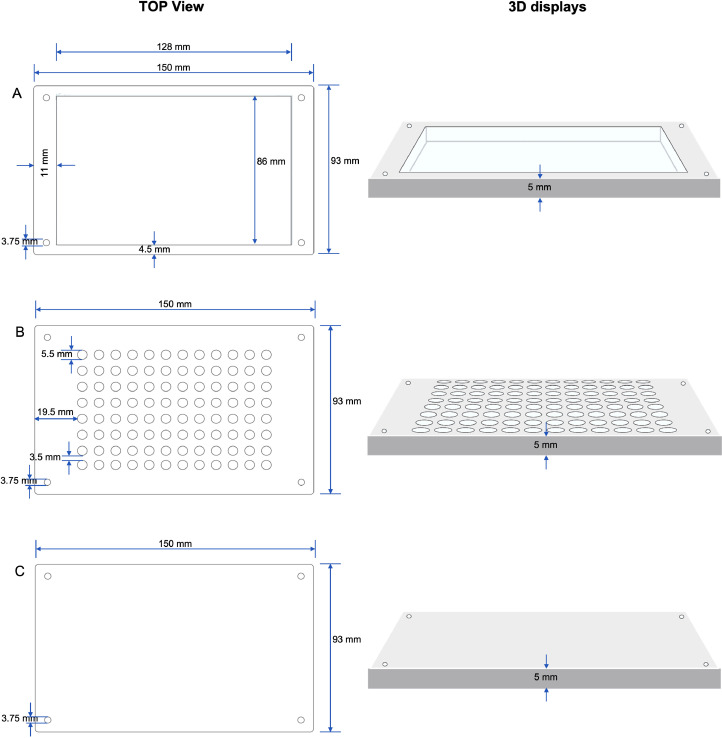
Design configurations of the components of the platform consisting of (A) Top POM sheet, (B) Middle POM sheet, and (C) Base POM sheet for the permanent magnets and 96-well plate.
  (2) Middle (Fig. 1B)
    • Designed to secure the permanent magnets.
    • Each compartment has a 5.5 mm diameter to accommodate each 5 mm permanent magnet.
    • The permanent magnet's 5 mm diameter, smaller than the well plate's bottom diameter (6.21 mm), compensates for the magnetic field strength of the magnet, which is most potent at its center and edges. This also provides a clear field of view for cell examination under the influence of magnets.
  (3) Bottom (Fig. 1C)
    • Works in conjunction with the middle layer to affix the magnet to its surface and maintain overall platform stability.
c. Translate or create the design images into a vector format.
  **Note**: Vector images can be enlarged without compromising the quality of the original image's lines and colors. A vector path with minimal line thickness is required by the laser machine.
d. Set the line width to “Hairline” and adjust the line color to RGB red.
e. Finally, export the completed design as a Scalable Vector Graphics (SVG) file.

a. Craft sketches for each component of the platform utilizing the graphic design software CorelDRAW.
  **Note:** If desired, an image file (JPG, PNG, or GIF) can be directly imported into the graphic design space of CorelDRAW as an alternative.
b. Select the dimensions for each component:
  (1) Top (Fig. 1A)
    • Engineered to snugly fit the lower body of the 96-well plate.
    • The design's thickness ensures sufficient clearance to avoid direct contact with the lid of the 96-well plate, thus eliminating potential contamination.

#### 2. Construction of the platform using a laser engraving machine

a. Position the POM sheet on the cutting table, having a working area of 300 × 200 mm^2^. Secure the material using the spring system attached to the working table. Make necessary adjustments to the table if required.
b. Within the CorelLASER software toolbar embedded in CorelDRAW, modify the settings as follows:
  • Resolution: 1000 dpi
  • Max speed: 500 mm/s
  • Page size (x-axis): 300 mm
  • Page size (y-axis): 200 mm
c. Define the file type that the program will interpret:
  • Cutting data: PLT-HP-GL/2 Plotter file
  • Cutting area: Current page
d. Select the “Cutting” mode of laser operation and adjust the parameters as listed below:
  • Speed = rate of laser shooting (5 mm/s)
  • Power = intensity of the laser (50 Watts)
  • Frame = shape of the frame (Rectangle)
  • Refer = relative position of the laser head (TopRight)
  • Refer-*X* = x coordinate of the laser head
  • Refer-*Y* = y coordinate of the laser head
e. Manually set the distance between the POM plate and the laser head to approximately 8 mm.
f. Click “Starting” to initiate the laser cutting process.
  **Note**: Prior to activating the laser operation, ensure that the air pump is turned on to avert burning the material due to generated heat. A few attempts may be necessary to find the optimal settings in the software for the material. Only change one operational condition at a time. For power, begin from a low value and gradually increase until the appropriate conditions are met.
g. Use a caliper to verify if the POM plate has the correct dimensions.

#### 3. Measurement of the magnetic field (B) of the permanent magnets on the empty 96-well plate using a Gaussmeter

**Fig. 2:**
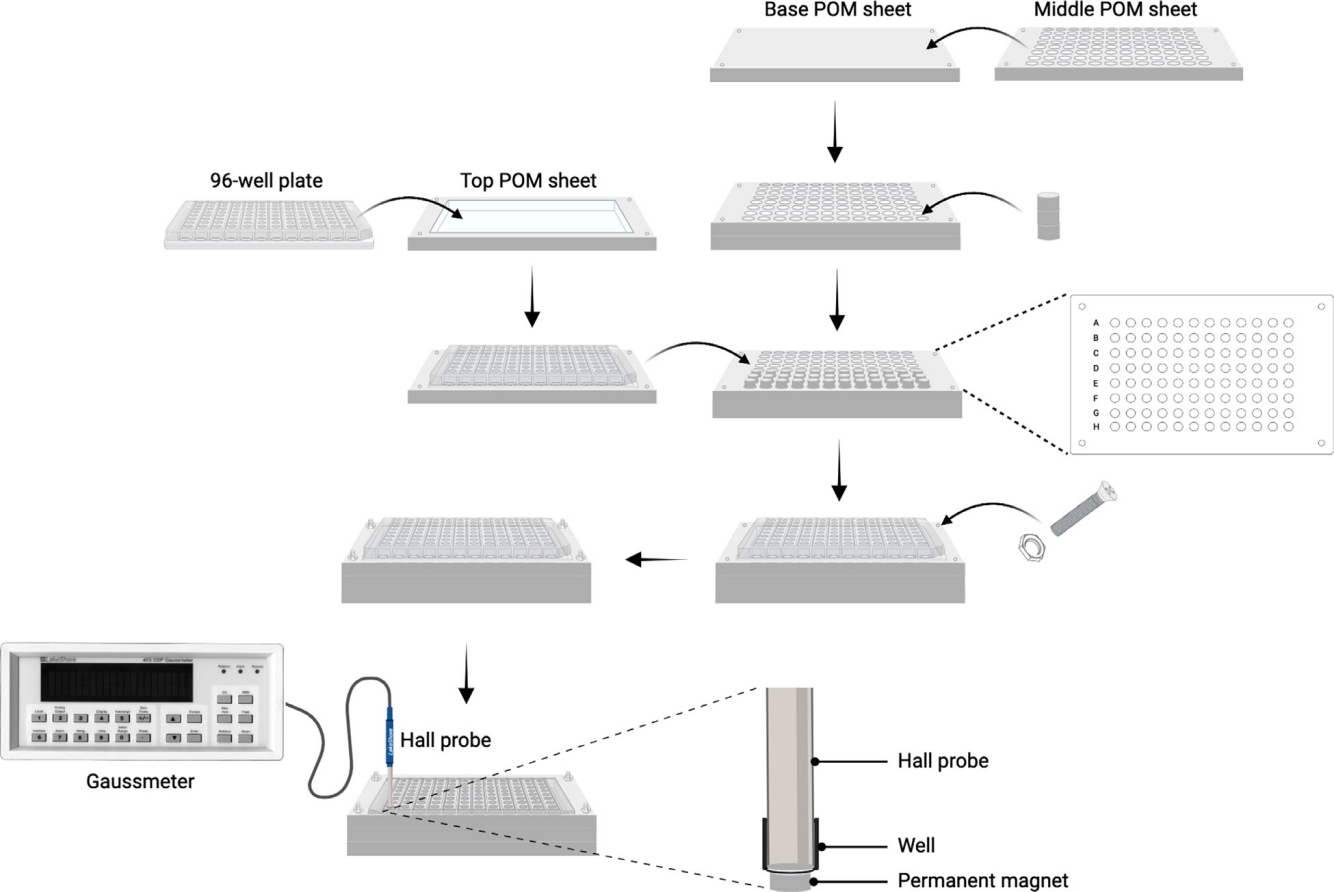
Complete assembly of the platform components with the installment of the permanent magnets and 96-well plate and measurement of the magnetic field.

**Note**: Lake Shore Cryotronics, Inc. has discontinued the supply of Model 455-DSP Gaussmeter and recommends using Model F71 instead. Small permanent magnets are typically used for biomedical applications and are classified as low field gradients [1]. A visual depiction of the platform assembly and the magnetic field measurement is shown in Fig. 2.

a. Allow the Gaussmeter and Hall probe to equilibrate at least 30 min before use.
b. Attach the 96-well plate to the top POM sheet.
c. Stack the middle POM sheet on top of the base POM sheet.
d. Place three permanent magnets (total length, 9 mm) in each compartment (letters F to H) of the middle POM sheet and secure them in place.
  **Note**: A process of trial and error is used to determine the positions of the permanent magnets on the 96-well plate. Compartments labeled A to C are used for experiments without permanent magnets. Compartments D and E are left vacant because positioning permanent magnets in these compartments results in magnetic field readings in compartments B and C, even without magnets present in these locations. Leaving compartments D and E empty ensures a reliable comparison of the nanoparticle (NP) cytotoxicity results between compartments A to C (cells treated in the absence of permanent magnets) and compartments F to H (cells treated in the presence of the permanent magnets).
e. Place the top layer (96-well plate + top POM sheet) over the stacked assembly (middle POM sheet with magnets + base POM sheet).
f. Carefully lift the entire assembly and secure all plates with the screws.
g. Place the fixed assembly onto a clean and uncluttered working surface.
h. Gently remove the lid of the 96-well plate.
i. Measure the B of each magnet by vertically positioning the Hall probe and attaching the round axial sensor to the bottom of the 96-well plate.
  **Note**: Maintaining the Hall probe perpendicular to the assembly's resting plane ensures a maximum reading output from the Gaussmeter (zero percent reading error). Additionally, a vertical distance (1.2 mm) exists between the internal well bottom (where cells attach) and the external well bottom (where the magnet directly contacts). Hence, B is not measured directly on the surface of the permanent magnet. The well bottom (diameter, 6.35 mm) suits the attachment of the Hall probe (diameter, 6.20 mm). This approach offers a realistic approximation of the actual B experienced by the NP-treated cells. The mean (standard deviation) B of the magnets used for the 96-well plates is 0.3149 (2.53) Tesla (*n* = 6).

## Fabrication of the CS/Alg-IONPs

### Materials

**Note**: The materials are listed in the sequence in which they appear in the procedure.

• Ultrapure water, Type 1
• Ultrafiltration system (MicroPure^Ⓡ^, Thermo Scientific, Germany)
• Graduated cylinder, 250 mL
• Erlenmeyer flask, 250 mL, 500 mL
• Magnetic bar, 40 × 8 mm^2^, 25 × 6 mm^2^
• Magnetic hotplate stirrer (ONiLAB^Ⓡ^, ONiLAB LLC Scientific Inc., CA, USA)
• Analytical balance (MC1, Sartorius, Gottingen, Germany)
• Sodium Alg powder (medium viscosity, Sigma-Aldrich Inc., St. Louis, MO, USA)
• Laboratory wrapping film (Parafilm^Ⓡ^ M, Bemis, Pittsburgh, PA, USA)
• Filter adapter, conical, rubber for Büchner filter funnel (Duran^Ⓡ^, DWK Life Sciences, NJ, USA)
• Filtering flask, 250 mL, 500 mL (Duran^Ⓡ^, DWK Life Sciences, NJ, USA)
• Büchner filter funnel, porcelain, 100 mL (United Scientific Supplies, Inc., Fisher Scientific, Pittsburgh, PA, USA)
• Glass microfiber membrane filter, Grade GF/C, 47 mm circle (Whatman^Ⓡ^, Cytiva, MA, USA)
• Vacuum pump (Gast, Gast Manufacturing Inc., MI, USA)
• Bottles, 100 mL, 500 mL (Duran^Ⓡ^, DWK Life Sciences, NJ, USA)
• Pipettor, 1000 µL (Eppendorf AG, Hamburg, Germany)
• Pipette tips, 1000 µL (Axygen^Ⓡ^, Corning Life Sciences Co., Wujiang, Jiangsu Province, China)
• Glacial acetic acid (Carlo Erba Reagents, Val de Reuil, Normandie, France)
• CS (MW ∼23 kDa; degree of deacetylation ∼91.0%, Marine Bio-Resources Co., Ltd., Samut Sakorn, Thailand)
• Incubator shaker (MaxQ 6000, Thermo Scientific, OH, USA)
• Sodium hydroxide pellets (Carlo Erba Reagents, Val de Reuil, Normandie, France)
• pH meter (SevenCompact, Mettler-Toledo, OH, USA)
• Ferrous chloride tetrahydrate (FeCl_2_·4H_2_O) (Acros Organics, NJ, USA)
• Ferric chloride hexahydrate (FeCl_3_·6H_2_O) (Carlo Erba Reagents, Val de Reuil, Normandie, France)
• Plastic spatulas
• Weighing containers
• Conical tubes, 50 mL (Corning CentriStar™, Corning Life Sciences Co., Wujiang, Jiangsu Province, China)
• Vortex mixer (Genie 2, Scientific Industries, Inc., NY, USA)
• Ultrasonic bath (Powersonic™, Crest Ultrasonics Corp., NJ, USA)
• Ice tubes
• Beaker, 150 mL capacity, 60 mm (diameter) × 80 mm (height) (Duran^Ⓡ^, DWK Life Sciences, NJ, USA)
• Nitrogen gas
• Rubber bands
• Nitrogen gas-filled balloon
• Hypodermic syringe, 10 mL, 20 mL (Nipro, Nipro Corp. Ltd, Thailand)
• Needle, #26 G, 1/2″ (Nipro, Nipro Corp. Ltd, Thailand)
• Syringe pump (New Era Pump Systems, Inc., USA)
• 30% Ammonia (Carlo Erba Reagents, Val de Reuil, Normandie, France)
• Permanent magnet, neodymium, 50 × 50 × 20 mm^3^ (Magnet Solution Co., Ltd, Nonthaburi, Thailand)
• AgNO_3_ powder (Carlo Erba Reagents, Val de Reuil, Normandie, France)
• pH-indicator strips (MQuant^Ⓡ^, Merck KGaA, Darmstadt, Germany)
• Lyophilizer (FreeZone, Labconco, MO, USA)

### Procedure

#### 1. Preparation of the sodium alginate (Alg) solution

Prepare a 0.2 mg/mL Alg solution with a total volume of 250 mL as follows:

**Note**: This volume is adequate for preparing four formulations.

a. Add 200 mL of ultrapure water to a 500 mL Erlenmeyer flask and place a magnetic bar (40 × 8 mm^2^) inside the flask.
b. Stir the water at 600 rpm and 45 °C for 15 min using a magnetic stirrer.
c. Weigh 50 mg of sodium Alg powder and gradually add it to the water. Then, cover the flask's opening with Parafilm^Ⓡ^ M while stirring continuously for 60 min.
d. Add ultrapure water to make a total volume of 250 mL.
e. Set up the filtration apparatus: Fit the filter adapter onto the opening of a 500 mL filtering flask. Secure the Buchner filter funnel on top of the adapter, then position the membrane filter at the center of the funnel.
f. Moisten the membrane filter and the funnel's interior with a small portion of the solution.
g. Carefully pour the solution against the sides of the funnel, avoiding direct contact with the membrane filter until the funnel is two-thirds full.
h. Turn the vacuum on, ensuring the membrane filter does not dry until all the solution is filtered.
i. Transfer the filtered solution to a 500 mL bottle and tightly close it.
j. Store the solution at 4 °C for up to 3 days.

#### 2. Preparation of the chitosan (CS) solution

Prepare a 0.23 mg/mL CS solution with a total volume of 50 mL as follows:

**Note**: This volume is adequate for preparing four formulations.

a. Prepare 100 mL of a 1% (*v/v*) acetic acid solution by diluting 1 mL of glacial acetic acid with 95 mL of ultrapure water. Add ultrapure water to achieve a total volume of 100 mL.
b. Pour 40 mL of the 1% (*v/v*) acetic acid solution into a 250 mL Erlenmeyer flask.
c. Weigh out 11.5 mg of CS powder and gradually add it to the acetic acid solution.
d. Cover the flask's opening with Parafilm^Ⓡ^ M and shake at 150 rpm for 24 h to ensure total dissolution of CS.
e. Adjust the pH of the CS solution to 5.0 using 2 M NaOH. Then, add ultrapure water to make a total volume of 50 mL.
  **Note:** Dissolve 0.8 g of NaOH pellets in ultrapure water to make 10 mL of 2 M NaOH. Let the solution cool to room temperature before use.
f. Set up the filtration apparatus: Fit the filter adapter onto the opening of a 500 mL filtering flask. Secure the Buchner filter funnel on top of the adapter, then position the membrane filter at the center of the funnel.
g. Moisten the membrane filter and the funnel's interior with a small portion of the solution.
h. Carefully pour the solution against the sides of the funnel, avoiding direct contact with the membrane filter until the funnel is two-thirds full.
i. Turn the vacuum on, ensuring the membrane filter does not dry until all the solution is filtered.
j. Transfer the filtered solution to a 500 mL bottle and tightly close it.
k. Store the solution at 4 °C for up to 3 days.

#### 3. Preparation of the solutions of FeCl_2_·4H_2_O and FeCl _3_·6H_2_O

Freshly prepare 50 mL of each FeCl_2_·4H_2_O and FeCl_3_·6H_2_O solution as follows:

**Note**: This volume is sufficient to make four formulations. The quantities of FeCl_2_·4H_2_O and FeCl_3_·6H_2_O are based on a stoichiometric mole ratio of 1:2. The total Fe precursor (mole) is 0.0010.

a. Weigh 0.2485 g of FeCl_2_·4H_2_O and 0.6760 g of FeCl_3_·6H_2_O.
  **Note**: Handle FeCl_2_·4H_2_O with care due to its oxidation sensitivity. Clean spatulas are necessary to prevent degradation of FeCl_2_·4H_2_O. FeCl_2_·4H_2_O crystals should be light green without any brown particles (FeO). Store FeCl_2_·4H_2_O and FeCl_3_·6H_2_O reagent bottles tightly sealed on a dry shelf or in a cabinet at 20–25 °C and away from natural light.
b. In separate 50 mL conical tubes, dissolve each Fe salt in 30 mL ultrapure water.
  **Note**: The synthesis of IONPs needs strict experimental conditions control. Using clean, dry 50 mL conical tubes helps to prevent unwanted reactions. An initial volume of 30 mL ensures adequate vortex mixing of the powder.
c. Seal each tube, vortex mix for 1 min, and sonicate the tubes at 9 W for 10 min while keeping the bath cool with ice.
d. Add 20 mL of ultrapure water to each tube, then vortex mix again.
  **Note**: Alternatively, invert the tubes 20 times for adequate mixing since blending a 50 mL solution in a 50 mL conical tube may need some pressure adjustment for efficient vortex mixer use. Use these solutions within 8 h.

#### 4. Preparation of the CS/Alg-IONPs

 The procedure for synthesizing CS/Alg-IONPs is adapted from previous research [2]. The quantities of Alg, CS, FeCl_2_·4H_2_O, and FeCl_3_·6H_2_O have been optimized to obtain desirable characteristics of the polymer-coated IONPs.
  **Note**: Set the syringe pump at a flow rate of 40 mL/h, with a syringe diameter of 20.12 mm, and a volume of 10 mL. Nitrogen gas is used to saturate the mixing vessels at all reaction stages. Mixing is performed at 1000 rpm at room temperature. All solutions should also be at room temperature when used.

a. Pour 55 mL of the Alg solution into a 150 mL beaker and place the magnetic bar (25 × 6 mm^2^) into the Alg solution.
  **Note**: Duran^Ⓡ^ beaker is made of highly inert borosilicate glass.
b. Purge the solution of dissolved oxygen by bubbling it with nitrogen gas for 10 min, then cover the beaker's opening with Parafilm^Ⓡ^ M and secure it with rubber bands.
c. Connect the nitrogen gas-filled balloon to the beaker to saturate it with nitrogen gas.
d. Fill the 20 mL syringe with 13 mL of FeCl_2_·4H_2_O solution, connect the needle carefully, and fasten the syringe to the syringe pump.
  **Note**: Before starting the formulation, prime the syringe pump by delivering a few drops of the solution to minimize delays and inaccuracies in the volume to be delivered. Ensure the plunger moves smoothly to maintain a steady flow of the liquid, as interruptions may occur if small particles sediment at the lower portion of the syringe and block the needle.
e. Place the syringe pump vertically with the syringe in the beaker by making a small hole in the Parafilm^Ⓡ^ M using the needle shaft. Position the needle shaft a few mm below the Parafilm^Ⓡ^ M, ensuring the lower end of the needle hub remains above the Parafilm^Ⓡ^ M. Securely position the syringe pump and adjust the mixer's height to achieve these measurements.
  **Note**: After setting up, perform a trial and error to ensure the magnetic bar is at the center of the vessel while mixing at 1000 rpm. This prevents liquid from splashing over the reaction vessel's walls.
f. Mix the solution at 1000 rpm for a total of 45 min.
  **Note**: After 15 min, the syringe pump delivers 10 mL of the FeCl_2_·4H_2_O solution. Then, disconnect the syringe pump from the reaction vessel, continue mixing for the next 30 min, regularly check for splashes, and keep the magnet's center of gravity within the reaction vessel. At this point, the nitrogen gas-filled balloon should still be connected to the reaction vessel.
g. After a total of 45 min of mixing, add 10 mL of FeCl_3_·6H_2_O solution to the mixture under the same conditions.
h. After mixing FeCl_2_·4H_2_O and FeCl_3_·6H_2_O, add 6 mL of 30% ammonia to adjust the pH to 10. Stir the mixture continuously at 1000 rpm for 30 min.
  **Note**: Black precipitates of Alg-coated IONPs (Alg-IONPs) form instantly upon adding ammonia.
i. Remove the Parafilm^Ⓡ^ M and the magnetic bar after mixing.
j. Place the beaker on the permanent magnet (50 × 50 × 20 mm^3^) to separate the Alg-IONPs from the dispersion.
  **Note**: During this step, the first decantation process will remove excess ammonia, which has a powerful and irritating odor. Wear goggles, nitrile gloves, and a face mask for protection. Additionally, keep the permanent magnet away from magnetic materials or metals that could be attracted to it.
k. Add 80 mL of ultrapure water to the Alg-IONPs, gently swirl to mix, and separate the Alg-IONPs by placing the beaker on the permanent magnet.
  **Note**: Let the Alg-IONPs settle for 2–3 min before discarding the supernatant. Repeat this process until the washings reach a pH of approximately 7.0. Also, check the washings for chloride ions using 1% (*w/v*) AgNO_3_. Four washing attempts are enough to achieve a neutral pH and ensure the absence of chloride ions in the dispersion.
l. Disperse the Alg-IONPs in ultrapure water to create a 50 mL dispersion, then place the magnetic bar in the mixture and cover the beaker's opening with Parafilm^Ⓡ^ M secured by rubber bands.
m. Mix the dispersion for 10 min, sonicate for 20 min, and then mix for another 10 min.
  **Note**: The pH of the dispersion ranges from 6.0 to 6.5, which keeps the carboxylate groups of Alg ionized in the mixture.
n. Connect the nitrogen gas-filled balloon to the beaker to saturate it with nitrogen gas.
o. Fill the 20 mL syringe with 13 mL of CS solution, then deliver 10 mL of the CS solution to the dispersion using the syringe pump with the same conditions and techniques.
  **Note**: The pH of the dispersion before the addition of CS should range from 6.0 to 6.5. After adding CS, the pH of the dispersion now ranges from 5.4 to 5.7.
p. Use the permanent magnet to separate the resulting CS/Alg-IONPs by attracting them to the beaker wall for 2 min. Then, carefully decant about 30 mL of the liquid from the solid NPs to remove the excess CS.
q. Pour the remaining mixture into a preweighed 50 mL conical tube, and collect the CS/Alg-IONPs by attracting them to the outside of the tube wall using a permanent magnet for 2 min. Afterward, carefully decant the liquid.
r. Freeze the CS/Alg-IONPs at –20 °C for 4 h and lyophilize at –80 °C for 24 h to obtain a dry powder.
s. Subtract the mass of the empty conical tube from the CS/Alg-IONP-filled tube to get the mass of the CS/Alg-IONPs.
  **Note**: A total of 50 to 55 mg of dry CS/Alg-IONP powder can be produced using this protocol.

## Cytotoxicity evaluation of the CS/Alg-IONPs toward MCF-7 breast cancer cells

### Materials

**Note**: The materials are listed in the sequence in which they appear in the procedure.

• Nitrile gloves (Sri Tang Gloves Co., Ltd., Pahtong, Hatyai, Songkhla, Thailand)
• Ultrapure water, Type 1
• Ultrafiltration system (MicroPure^Ⓡ^, Thermo Scientific, Germany)
• Graduated cylinder, 1000 mL
• Beaker, 1000 mL (Pyrex^Ⓡ^, DWK Life Sciences GmbH, Wertheim, Germany)
• 70% *(v/v)* ethanol
• Dulbecco's Modified Eagle Medium powder with L-glutamine, pyridoxine hydrochloride, and 110 mg/L sodium pyruvate (DMEM, Fisher Scientific, Pittsburgh, PA, USA)
• Magnetic Hotplate stirrer (ONiLAB^Ⓡ^, ONiLAB LL7C Scientific Inc., CA, USA)
• Sodium bicarbonate (NaHCO_3_, Ajax Finechem, Thermo Fisher Scientific, NSW, AUS)
• pH meter (SevenCompact, Mettler-Toledo, OH, USA)
• 1 M HCl
• Pipettor, 1000 µL (for acids/bases)
• Pipette tips, 1000 µL (Axygen^Ⓡ^, Corning Life Sciences Co., Wujiang, Jiangsu Province, China)
• Biological safety cabinet (BSC), Class A2 (Model 1386, Thermo Fisher Scientific, OH, USA)
• Bottles, 500 mL, 1000 mL (Duran^Ⓡ^, DWK Life Sciences, NJ, USA)
• Bottle top vacuum filter, 0.22 µm (Corning^Ⓡ^, Corning Incorporated – Life Sciences, AZ, USA)
• Vacuum pump (Gast, Gast Manufacturing Inc., MI, USA)
• Pipette controller (Falcon^Ⓡ^, Corning Incorporated – Life Sciences, NC, USA)
• Sterile pipettes, 2 mL (Nunc™, Thermo Fisher Scientific, Seoul, Korea)
• Sterile pipettes, 5 mL (Costar^Ⓡ^ Stripette^Ⓡ^, Corning Incorporated – Life Sciences, ME, USA)
• Sterile pipettes, 10 mL (Falcon^Ⓡ^, Corning Incorporated – Life Sciences, NC, USA)
• Fetal bovine serum (FBS, PAN-Biotech, Aidenbach, Germany)
• Water bath (DT Hetotherm^Ⓡ^, Heto-Holten, Allerd, Denmark)
• 10,000 Units/mL penicillin/10,000 µg/mL streptomycin (PenStrep, Gibco, Life Technologies, ON, Canada)
• Conical tubes, 15 mL, 50 mL (Corning CentriStar™, Corning Life Sciences Co., Wujiang, Jiangsu Province, China)
• Laboratory wrapping film (Parafilm^Ⓡ^ M, Bemis, Pittsburgh, PA, USA)
• Refrigerator
• Phosphate-buffered saline powder (PBS, Sigma-Aldrich^Ⓡ^, St. Louis, MO, USA)
• Sterilizer, top load (HVA 110 Hirayama Manufacturing Corp., Saitama, Japan)
• Analytical balance (MC1, Sartorius, Gottingen, Germany)
• 3-(4,5-dimethylthiazol-2-yl)−2,5-diphenyl tetrazolium bromide powder (MTT, (BD Pharmingen, San Diego, CA)
• Plastic spatula
• Weighing container
• Vortex mixer (Genie 2, Scientific Industries, Inc., NY, USA)
• Ultrasonic bath (Powersonic™, Crest Ultrasonics Corp., NJ, USA)
• Ice tubes
• Eppendorf tubes, 1.5 mL
• Aluminum foil
• MCF-7 human breast cancer cells (ATCC, Manassas, VA, USA)
• Centrifuge, refrigerated (Hermle Z 383 K, InProcess Instruments GmbH, Bremen, Germany)
• Cell culture flask, U-shaped, 75 cm^2^ growth area (Corning™, Corning Life Sciences Co., Wujiang, Jiangsu Province, China)
• CO_2_ incubator (Forma Series II Water Jacket, Model 3111, Thermo Fisher Scientific, OH, USA)
• Gas tanks with CO_2_
• 0.25% trypsin-EDTA (Gibco, Life Technologies, ON, Canada)
• Inverted microscope
• Coverslips
• Hemocytometer (Boeckel + Co (GmbH + Co), Hamburg, Germany)
• Trypan blue
• 96-Well, Clear, Flat-Bottom Polystyrene TC-Treated Microplate (Costar^Ⓡ^, Corning Life Sciences, Wujiang, Jiangsu Province, China) Product No. 3599
• Pipettors, 100 µL, 1000 µL (Eppendorf AG, Hamburg, Germany)
• Pipette tips, 200 µL, 1000 µL (Axygen^Ⓡ^, Corning Life Sciences Co., Wujiang, Jiangsu Province, China)
• Small plastic bags
• Permanent magnets, N35 NdFeB round magnets, 5 × 3 mm^2^ (diameter × length) (Ningbo Townsun Magnet Co., Gaoqiao Industrial Development Area, Yinzhou District, Ningbo, China)
• CSK Philip machine screws, 2 × 22 mm^2^ (diameter × length), and hex lock nut
• POM assembly
• Multichannel pipettor (Axygen^Ⓡ^ Axypet^Ⓡ^, Corning Incorporated – Life Sciences, AZ, USA)
• Dimethylsulfoxide (DMSO, Emplura^Ⓡ^, Merck KGaA, EMD Millipore Corp., Darmstadt, Germany)
• Microplate reader (CLARIOstar, BMG Labtech GmbH, Germany)

### Procedure

#### 1. Preparation of basal growth medium

 Prepare 1000 mL of basal medium (DMEM) at room temperature as follows:
  **Note**: The procedures from steps a to e can be conducted nonsterile. Always put on nitrile or latex-style gloves when handling materials meant for cell culture.

a. Measure 1000 mL of ultrapure water using a 1000 mL graduated cylinder and pour 800 mL into a 1000 mL beaker.
b. Dissolve a sachet of DMEM powder in the 800 mL of ultrapure water in the beaker under magnetic stirring.
c. Add 3.7 g of NaHCO_3_ to the beaker and continue stirring until the powder is completely dissolved.
d. Measure the pH of the solution and adjust it by adding 1 M HCl dropwise until the pH reaches 7.2.
e. Add more ultrapure water to the beaker to make up a final volume of 1000 mL.

**Note:** Perform all subsequent operations inside the BSC. Before use, sanitize the BSC with the UV lamp for 30 min and clean it with 70% ethanol. Also, sterilize all materials with 70% ethanol before placing them inside the BSC.

a. Attach the bottle top vacuum filter to the pre-sterilized 500 mL bottle and connect the vacuum pump hose to the filter.
b. Gradually pour 500 mL of the medium along the sides of the filter.
c. Turn the vacuum on and filter the medium.
d. Close the bottle with its cap.
e. Repeat the process to filter the remaining 500 mL medium.
  **Note**: 500 mL of serum-free DMEM is used to prepare the complete DMEM, while the remaining 500 mL of serum-free DMEM serves as a stock and diluent for preparing the NP treatment samples

#### 2. Preparation of the complete growth medium

 Prepare 500 mL of complete growth medium (complete DMEM) as follows:
  **Note:** Perform all subsequent operations inside the BSC. Before use, sanitize the BSC with the UV lamp for 30 min and clean it with 70% ethanol. Also, sterilize all materials with 70% ethanol before placing them inside the BSC.

a. Measure 445 mL of the freshly-prepared serum-free DMEM in a 500 mL sterile bottle.
b. Add 50 mL heat-inactivated FBS using a sterile pipette and pipette controller.
  **Note**: The FBS should be pre-heat-inactivated at 56–58 °C for 30 min in a water bath.
c. Add 5 mL of PenStrep (1% *v/v*) using a sterile pipette and pipette controller and gently swirl the solution to avoid bubble formation.
d. Transfer 40 mL of the complete DMEM to 50 mL conical tubes and seal with Parafilm^Ⓡ^ M. Store the medium at 4 °C until use.

#### 3. Preparation of the phosphate-buffered saline (PBS) solution

Prepare 1000 mL of PBS at room temperature as follows:

**Note**: The procedures from steps a to g can be conducted nonsterile.

a. Measure 1000 mL of ultrapure water using a 1000 mL graduated cylinder and pour it into a 1000 mL beaker.
b. Add the PBS powder to the water while stirring until it completely dissolves.
c. Transfer the solution to a 1000 mL bottle with a slightly loosened cap.
d. Sterilize the solution in an autoclave at 121 °C for 20 min.
e. Close the cap and allow the solution to cool to room temperature.
f. Transfer 40 mL of PBS to 50 mL conical tubes inside the BSC, seal with Parafilm^Ⓡ^ M and store at 4 °C until use.
g. Secure the cap of the remaining PBS in the 1000 mL bottle with Parafilm^Ⓡ^ M and store at 4 °C until use.

#### 4. Preparation of the MTT stock solution

Prepare the MTT stock solution as follows:

a. Weigh 50 mg of the MTT powder and place it in a 15 mL conical tube.
b. Dissolve the powder with 1 × PBS to make 10 mL of 5 mg/mL MTT solution using a vortex mixer for 1 min.
c. Sonicate the solution for 5 min while keeping the tube in an ice bath.
d. Make 1 mL aliquots using 2 mL Eppendorf tubes, wrap each tube in foil, and freeze at –20 °C until use [3].

#### 5. Cell cultivation

**Note**: Ensure all media are pre-warmed to 37 °C in a water bath, and always use a pipette controller for all pipetting operations.

a. Thaw the tube containing frozen MCF-7 cells within 2 min in a water bath at 37 °C with gentle shaking.
b. Disinfect the outside of the tube with 70% ethanol and place it in the BSC.
c. Transfer the thawed 1 mL cell suspension to a 15 mL conical tube, slowly adding 9 mL of pre-warmed complete DMEM.
d. Mix the cell suspension and complete DMEM gently.
e. Centrifuge the cell suspension at 130 × g at 4 °C for 5 min.
f. Discard the supernatant and gently resuspend the cell pellet in 13 mL of complete DMEM in a 75 cm^2^ flask.
g. Incubate the cells at 37 °C in a humidified environment with 5% CO_2_.
h. After 24 h, replace the medium with an equivalent volume of fresh complete DMEM.
i. Allow the cells to grow to 85% confluency, which usually takes 3–4 days, with medium replacement every 2 days.
j. Once the cells reach confluency, remove the medium employing a 10 mL sterile pipette, and wash the cells by adding 8 mL of PBS using a sterile pipette.
  **Note**: To avoid disturbing the adherent cell monolayer, gently pour the PBS down the flask's wall. Allow the PBS to cover the cell monolayer completely by tilting the cell culture flask in different directions for 1 min.
k. Remove the PBS washing and trypsinize the cells by adding 2 mL of trypsin.
  **Note**: Allow the trypsin to cover the cell monolayer by gently tilting the cell culture flask back and forth in opposing directions for 1 min.
l. Remove the trypsin, secure the cap, and incubate the cells for 2 min.
m. Add 8 mL of complete DMEM to the cells to stop the trypsin activity.
  **Note**: During this step, the cells may become visible and flow freely at the bottom of the flask.
n. Detach the cells from the flask's bottom using reverse and forward pipetting techniques.
  **Note**: Avoid expelling all the liquid during forward pipetting to prevent excessive bubble formation. Fill the pipette with up to 5 mL for reverse pipetting to maintain the hydrodynamic flow without disruption. Ensure the pipette's tip is submerged in the liquid but not in contact with the flask's surface.
o. Retain 2 mL of the cell suspension in the cell culture flask and use the remaining 6 mL for cell seeding.
p. Resuspend the cells by adding 11 mL of complete DMEM and mix gently.
q. Observe the cells under a microscope.
  **Note**: The cells should appear as individual entities floating freely. If aggregation is observed, gently use the reverse and forward pipetting techniques to disperse them.
r. Incubate the cells at 37 °C in a humidified environment with 5% CO_2_.
s. Refresh the medium with a new complete DMEM every two days.
t. Grow the cells to 85% confluency before proceeding with subsequent experiments.

#### 6. Cell seeding

**Note**: To avoid thermal shock, all media should be preheated to 37 °C in a water bath.

a. Proceed with the cell seeding on the 96-well plate once cell confluency reaches 85%.
b. Place 6 mL of cell suspension from the cultivation step in a 50 mL conical tube.
c. Prepare the hemocytometer for cell counting in the BSC.
  **Note**: Clean the hemocytometer and its coverslip with a solution of 70% ethanol.
d. Dispense 25 µL of Trypan blue into an Eppendorf tube.
e. Transfer 25 µL of the cell suspension into the same Eppendorf tube.
f. Achieve an even mix through a cycle of reverse and forward pipetting.
g. Extract 10 µL from the mixed solution and deposit it in the hemocytometer.
  **Note**: Make sure to count the viable (unstained) cells within a 3–5 min timeframe to avoid compromising cell viability [3].
h. Compute the average cell count from the four quadrants of the hemocytometer.
i. Prepare 12 mL cell suspension for a 96-well plate at an optimal cell concentration of 10,000 cells/100 µL/well.
j. Determine the volume of the cell suspension to be retrieved from the 50 mL conical tube.
k. Ascertain the complete DMEM volume by deducting the cell suspension volume from 12 mL.
l. Introduce the necessary amount of complete DMEM into an unused 50 mL conical tube.
m. Add the computed volume of the cell suspension to the tube.
n. Securely close the tube and gently agitate the cell suspension in a pendulum-like motion, holding the tube between your thumb and index finger.
  **Note**: Refrain from using a vortex mixer to prevent cell aggregation in the center of the suspension.
o. Consistently hold the tube between the thumb and index finger while dispensing 100 µL of the cell suspension into each well of a 96-well plate.
  **Note**: With each addition, ensure gentle mixing of the cell suspension. As an alternative, a multichannel pipettor can be used. However, the process should be expedited to prevent the cells from settling at the reservoir's bottom.
p. Subject the cells to incubation at 37 °C in a CO_2_ incubator for 24 h.
q. Discard any remaining cell suspension in a bleach-filled waste container.

#### 7. Preparation of the CS/Alg-IONPs working suspensions

**Note:** Ensure that the CS/Alg-IONP samples are prepared no more than 60 min before the cell treatment.

a. Prepare a 6 mL stock solution of 2 mg/mL of freeze-dried CS/Alg-IONPs in serum-free DMEM, which can be used for one 96-well plate.
b. Weigh 12 mg of CS/Alg-IONP powder into a small plastic bag.
c. Sterilize the powder for 15 min using the UV lamp in the BSC.
d. Transfer the sterilized powder into a 15 mL conical tube.
e. Pour in an adequate quantity of serum-free DMEM to make 15 mL.
f. Mix the suspension using a vortex mixer for 30 s and further sonicate for 5 min.
g. Calculate the required volume of nanoparticle suspension to be taken from the stock solution for each desired concentration of the working suspension (1, 2, 4, 8, 16, 32, 64, 100% (*v/v*)).
  **Note**: Set aside 2 mL for each concentration. Each well should contain 0.2 mL of the CS/Alg-IONP suspension. For every concentration in a single 96-well plate, 6 wells are required, each containing 3 wells for treatment in the presence and absence of permanent magnets. A 2 mL volume allows for extra working suspension. Using serum-free DMEM, adjust each working suspension to the correct volume. Each working suspension should be prepared in a 15 mL conical tube.
h. Thoroughly mix the suspension using a vortex mixer.

#### 8. Treatment of the MCF-7 cells with the CS/Alg-IONP suspension

**Note**: The cells should be treated with the samples after 24 h of seeding. All materials (POM plates, screws, and magnets) should be sterilized with 70% ethanol and exposed to UV light for 30 min before assembling the platform and cell-seeded 96-well plate. Assemble the materials within the BSC.

a. Assemble the 96-well plate seeded with MCF-7 cells and the platform as demonstrated in Fig. 2A-E.
b. Place the assembled structure on a clean, uncluttered work surface within the BSC.
c. Remove the lid from the 96-well plate and remove the old medium from each well.
d. Add 0.2 mL of the working suspension to each well.
  **Note**: Before adding the working suspension to each well, perform the reverse and forward pipetting techniques to mix the CS/Alg-IONP suspension twice.
e. Gently lift the entire assembly and incubate the cells for 1 h at 37 °C in a humidified atmosphere with 5% CO_2_ by placing the assembly inside the incubator.
f. After 1 h, disassemble the platform from the 96-well plate within the BSC and continue incubating the cells (in the 96-well plate) for the next 23 h.
  **Note**: To compare the effect of incubation time, the cells can also be incubated in the presence of permanent magnets for 2, 4, or 6 h.

#### 9. MTT assay

Refer to a previous publication [3] for the MTT assay protocol. This custom method is designed specifically for handling IONPs.

a. After incubating the treated cells for 24 h, remove the medium, and rinse the cells twice with 0.2 mL of PBS.
  **Note**: The control (cells treated with the serum-free DMEM) should also be rinsed twice with PBS to account for the effect of rinsing on cell attachment. For each rinsing step, the PBS wash should be immediately pipetted out to prevent the settling of nanoparticles that may affect absorbance readings.
b. Prepare a 0.5 mg/mL MTT solution by adding 0.7 mL of MTT stock solution to 6.3 mL of serum-free DMEM in a 15 mL conical tube, creating a 7 mL MTT solution. Vortex mix the solution. Protect the solution from light.
c. Add 0.1 mL of the MTT solution to each well and incubate the cells at 37 °C for 2 h.
d. Remove the MTT medium.
e. Add 0.1 mL of DMSO to each well and gently shake the plate to dissolve the formazan crystals.
f. Measure the optical density at 570 nm using a microplate reader.
g. Calculate the cell viability (%) using:
  $$\text{Cell}\;\text{viability}\;\left(\%\right)=\frac{N_{t}}{N_{c}}\times \;100$$
  where N_t_ and N_c_ represent the optical densities of the treated and untreated (control) cells, respectively.

## Declaration of generative AI and AI-assisted technologies in the writing process

During the preparation of this work, the authors used ChatGPT 4.0 in order to improve language and readability. After using this tool, the authors reviewed and edited the content as needed and take full responsibility for the content of the publication.

## CRediT authorship contribution statement

**Bryan Paul Bulatao:** Conceptualization, Methodology, Visualization, Formal analysis, Investigation, Writing – original draft. **Nonthaneth Nalinratana:** Conceptualization, Methodology, Visualization, Writing – review & editing. **Pongsakorn Jantaratana:** Conceptualization, Methodology, Visualization. **Opa Vajragupta:** Supervision, Conceptualization, Writing – review & editing. **Pornchai Rojsitthisak:** Supervision, Conceptualization, Writing – review & editing. **Pranee Rojsitthisak:** Supervision, Conceptualization, Writing – review & editing, Funding acquisition, Project administration.

## Data Availability

Data will be made available on request.
